# High-Dimensional Fixed Effects Profiling Models and Applications in End-Stage Kidney Disease Patients: Current State and Future Directions

**DOI:** 10.6000/1929-6029.2023.12.24

**Published:** 2023-02-15

**Authors:** Danh V. Nguyen, Qi Qian, Amy S. You, Esra Kurum, Connie M. Rhee, Damla Senturk

**Affiliations:** 1 Department of Medicine, University of California Irvine, Orange, CA 92868, USA; 2 Department of Biostatistics, University of California, Los Angeles, CA 90095, USA; 3 Department of Statistics, University of California, Riverside, CA 92521, USA; 4 Department of Medicine, University of California, Los Angeles, CA 90095, USA; 5 VA Greater Los Angeles Medical Center, Los Angeles, CA 90073, USA

**Keywords:** Dialysis facility staffing, end-stage kidney disease, fixed effects, generalized linear mixed model, high-dimensional parameters, multilevel varying coefficient model, Poisson regression, propensity score, random effects, United States Renal Data System

## Abstract

Profiling analysis aims to evaluate health care providers, including hospitals, nursing homes, or dialysis facilities among others with respect to a patient outcome, such as 30-day unplanned hospital readmission or mortality. Fixed effects (FE) profiling models have been developed over the last decade, motivated by the overall need to (a) improve accurate identification or “flagging” of under-performing providers, (b) relax assumptions inherent in random effects (RE) profiling models, and (c) take into consideration the unique disease characteristics and care/treatment processes of end-stage kidney disease (ESKD) patients on dialysis. In this paper, we review the current state of FE methodologies and their rationale in the ESKD population and illustrate applications in four key areas: profiling dialysis facilities for (1) patient hospitalizations over time (longitudinally) using standardized dynamic readmission ratio (SDRR), (2) identification of dialysis facility characteristics (e.g., staffing level) that contribute to hospital readmission, and (3) adverse recurrent events using standardized event ratio (SER). Also, we examine the operating characteristics with a focus on FE profiling models. Throughout these areas of applications to the ESKD population, we identify challenges for future research in both methodology and clinical studies.

## 1. INTRODUCTION

The latest national data from the United States Renal Data System (USRDS) shows that about 130,500 ESKD patients transitioned to dialysis in 2020 in the United States, and, furthermore, for nearly a decade (since 2014) over 120,000 individuals with ESKD transition to dialysis annually [1]. Unlike patients with many other chronic diseases, individuals with ESKD require long-term renal replacement therapy (dialysis) to sustain life due to kidney failure.

There are distinct characteristics as well as treatment processes unique to ESKD patients. For example, ESKD patients treated with dialysis have a high burden of complex comorbid conditions and patients experience frequent hospitalizations over time (about twice per year) with hospitalization rates markedly elevated and 30-day readmission is about 31%, which is twice the rate of readmission seen in older Medicare beneficiaries without kidney disease [2]. In the first year after transition to dialysis treatment, mortality rates are also very high [3–8] and even among ESKD patients who survive this fragile transition period survival rates are substantially low (5-year survival < 40%) which is worse than Medicare populations (age 65+) with heart failure, acute myocardial infarction, and cancer [9]. With respect to treatment processes, patients on dialysis require “continuous” medical care on maintenance dialysis due to the limited treatment options through kidney transplantation. They receive dialysis care at over 6,000 dialysis facilities, typically dialyze three times per week, and have regular interactions with nephrologists, patient care technicians (PCT), nurses, dietitians, and other dialysis facility staff. Furthermore, they are monitored regularly with respect to targeted patient outcomes, including dialysis adequacy (to ensure sufficient removal of waste from blood); bone and mineral disorder (e.g., to prevent high calcium in the blood or hypercalcemia); phosphorous level; regulation of blood pressure; and hemoglobin (Hb) to manage anemia among other conditions.

In addition to the aforementioned unique treatment processes and management to ensure quality of care for patients with ESKD, frequent hospitalizations (especially unplanned hospital readmission) and mortality clearly impacts patients’ quality of life, remaining lifespan, and healthcare cost. For example, although ESKD accounts for less than 2% of the Medicare beneficiaries, ESKD consumes about 7% (about $50 billions in 2018) of total Medicare budget [1].

Therefore, profiling analysis or assessment of dialysis facilities’ performance with respect to key patient outcomes, including mortality, hospitalization, readmission, and adverse events provides metrics of safe and adequate delivery of health care to patients. This evidence-based approach to measure performance is consistent with national objectives to “improve support for a culture of safety” and “reduce inappropriate and unnecessary care that can lead to harm” [11]. As described above, unique aspects of the ESKD population include frequent hospitalization, periods of high mortality after transition to dialysis, and continuous care and monitoring of adverse events. Thus, tailored profiling methods have been proposed to incorporate these patient and population characteristics.

The remainder of our paper is organized as follows. In Section 2, we summarize the evolving literature on profiling models, starting with random effects (RE) profiling models and leading to fixed effects (FE) profiling models developed over the last decade along with their motivations which diverge from RE models. In Section 3 and Section 4, we describe the methods and illustrative applications to profiling dialysis facilities (1) using time-dynamic profiling (TDP) for a longitudinal outcome (hospitalization), (2) for identification of dialysis facility characteristics (e.g., staffing level) that contribute to hospital readmission, and (3) for adverse recurrent events. In Section 5, we describe studies examining the operating characteristics of FE models, including performance under sparse outcomes (e.g., cause-specific hospitalization), the impact of inadequate case-mix adjustment, and comparisons with RE models. For each area, we discuss challenges for future research. We conclude with a discussion in Section 6.

## 2. PROFILING MODELS AND LITERATURE

The objectives of profiling analyses are multifaceted and include 1) identifying providers (e.g., hospitals, dialysis facilities, nursing homes etc.) with performance below standard by government agencies for regulatory or payment purposes; 2) conveying information to patients regarding the quality of care of providers; and 3) providing feedback to providers for quality improvement among others. Although profiling dates back nearly a century [12], more systematic reporting of patient outcomes among providers has only appeared directly to consumers in the last decade by CMS. This includes condition-specific 30-day mortality (e.g., acute myocardial infarction [MI], heart failure, pneumonia) and 30-day (all-cause) readmission rates; see Keenan *et al*. (2008), Krumholz *et al*. (2011), Lindenauer *et al*. (2011), Ross *et al*. 2010, Horwitz *et al*. (2011) and Horwitz *et al*. (2014) [13–18]. Typically, profiling of providers, such as hospitals are performed annually, especially with respect to objective 1.

### 2.1. RE Profiling Models and Literature

The main goals of profiling analysis are to provide estimates of provider effects, relative to a reference norm, and to identify/flag providers’ performances that are extreme, i.e., significantly worse than the reference norm (under-performing). For example, in assessing the rate of 30-day (unplanned) hospital readmission, the objectives are to (1) provide reliable estimate of 30-day standardized readmission rate for each provider (i.e., hospital) and (2) flag providers whose performances are significantly below the reference rate, such as a national average/median rate. Profiling analysis has two key aspects. First, variation in patient outcomes, such as 30-day readmission and mortality, are modeled as a combination of variation in provider quality of care (provider effects) and variation in patient case-mix (patient-level factors including demographics, comorbidities, and types of index hospitalization). Furthermore, since patients are nested within providers, profiling models are hierarchical regressions of the form *outcome* = *provider effects + patient case-mix effects*. In applications to the general population, often the binary outcome is sparse since 30-day all-cause (as well as cause-specific or condition-specific such as myocardial infarction [MI], heart failure, etc.) hospitalization and mortality are “rare” relative to the total number of hospitalization events in a given year across the US. For these aforementioned aspects, the following hierarchical logistic regression RE profiling model have been proposed by Normand, Glickman and Gatsonis (1997) [19]:

(1) $$g\left\{E\left(Y_{ij}|Z_{ij}\right)\right\}\equiv g\left(\mu_{ij}\right)=\gamma_{i}+Z_{ij}^{T}\beta ,$$

where the provider effects $\gamma_{i}$ ’s are modeled as $\gamma_{i}\sim N\left(\gamma_{0},\sigma^{2}\right),\;i=1,\ldots ,I$ indexes providers (e.g., hospitals, dialysis facilities, etc.), $j=1,\ldots ,N_{i}$ indexes patients in provider $i,Y_{ij}$ is the outcome for patient j in provider $i,Z_{ij}={\left(Z_{1ij},\ldots ,Z_{rij}\right)}^{T}$ denotes the vector of r patient-level risk adjustment factors with parameters $\beta ={\left(\beta_{1},\ldots ,\beta_{r}\right)}^{T}$, and g(⋅) is a link function. For example, with 30-day hospital readmission, $\mu_{ij}=p_{ij}=\text{Pr }\left(Y_{ij}=1|\beta ,\gamma_{i},Z_{ij}\right)$ is the expected readmission for patient index discharge j in hospital i, and the link function is the logit link: $g\left(p_{ij}\right)=\text{log}\left\{p_{ij}\left(1-p_{ij}\right)\right\}$.

We note that in addition to the nested structure of the data, the sparse outcome data have naturally led to the adoption of modeling provider effects as random effects since RE models can provide stable provider effect estimates through shrinkage. For a more thorough example of the issues in profiling and historical perspectives, the reader is referred to a discussion of the statistical and clinical aspects of hospital outcomes profiling [20], issues in identifying unusual performance in health care providers with an example from the UK National Health Service (NHS) [21, 22], and early uses and perspectives on performance indicators [23].

**Figure 1:**
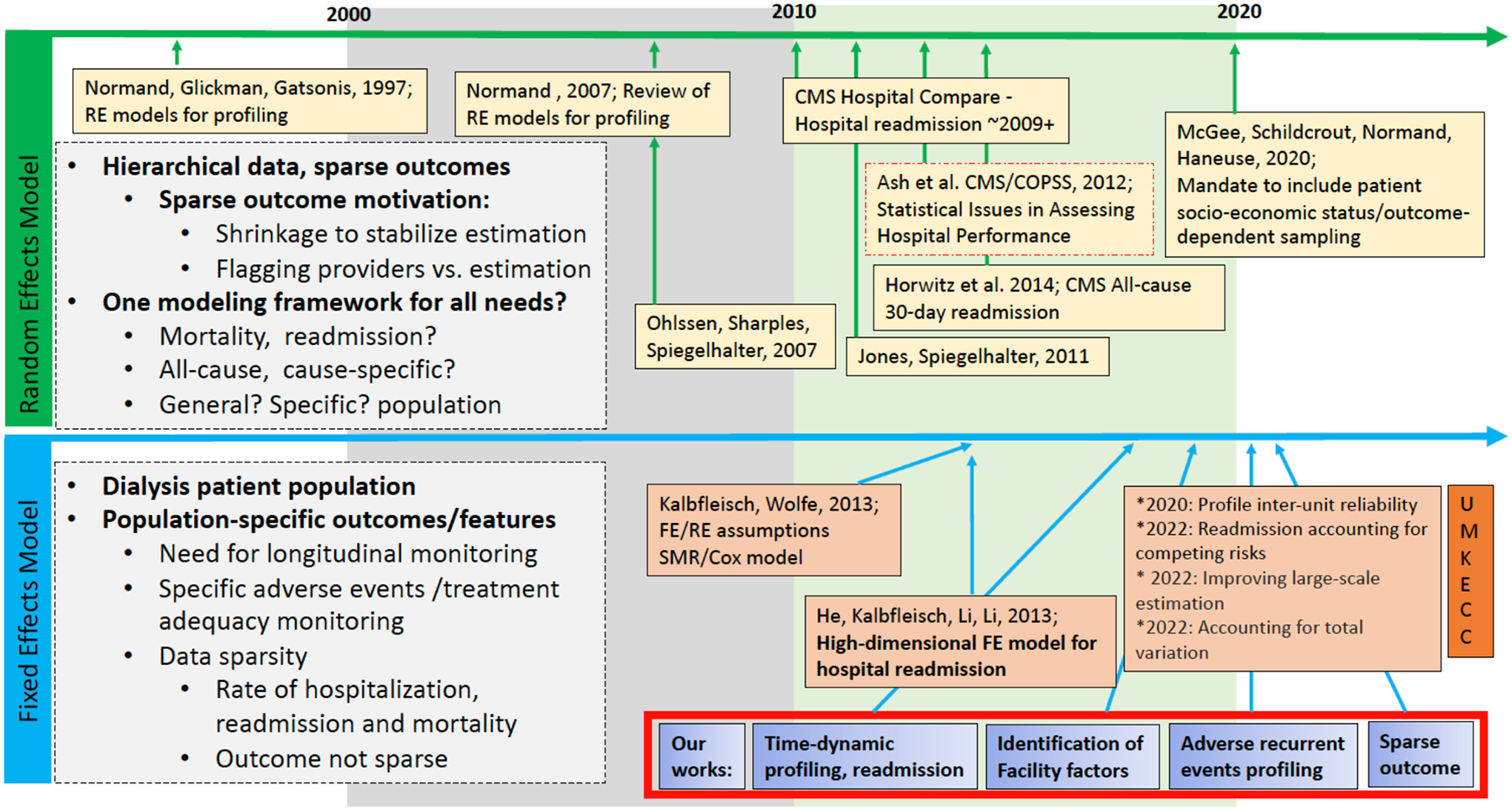
Selected main literature on profiling methodology development and applications over past two decades for random effects (RE) profiling model (top panel – green) and fixed effects (FE) profiling models focused on/tailored to unique aspects of end-stage kidney disease (ESKD) patients. Key works for RE models include Norman, Glickman and Gatsonis (1997) [19], Ash *et al*. (2012) [24], and Centers for Medicare & Medicaid Services (CMS) launch of “Hospital Compare” in ~2009 and for FE models this include the seminal works of Kalbfleisch and Wolfe (2013) [30] and He *et al*. (2013) [38] at the University of Michigan Kidney Epidemiology and Cost Center (UM-KECC). Our works also focused on profiling dialysis facilities and are highlighted in the red box and described in this paper.

In the US during the early 2010’s, the RE profiling model (1) was adopted by CMS (Hospital Compare; e.g., see [14–18]) and the appropriateness of the approach, including advantages and disadvantages, was assessed in the white paper, “Statistical issues in assessing hospital performance,” [24] by the Committee of Presidents of Statistical Societies (COPSS). The body of literature on profiling analysis, building onto the RE models (1), continued to evolve and includes works accounting for recent regulation mandating inclusion of socio-economic status [25]; incorporating more flexible hierarchical Bayesian RE models [26] and extending the CMS model (1) to incorporate provider characteristics (e.g., hospital volume, infrastructure etc.) and to improve prediction for mortality using performance metrics based on direct standardization [27, 28]. Figure 1 (top panel) displays the selective key literature on RE profiling models starting with the key paper by Norman, Glickman and Gatsonis (1997) [19], implementation in Hospital Compare [29] by CMS in the early 2010’s [17] and continued methodology development into the current time period (2020s’) [25].

### 2.2. Development of High-Dimensional FE Models for ESKD Population and Literature

As described in the previous section and summarized in Figure 1 (top panel - green), much of the literature on RE hierarchical profiling models have been developed over past 15 years with renewed interests and developments since 2010, following CMS launch of Hospital Compare in ~ 2009. A justification for the use of RE models is that they provide stable provider effect estimates through shrinkage [24], although several inherent disadvantages have been extensively discussed. Specifically, RE estimates are biased toward the overall provider average and biased in the presence of confounding between patient risk factors and provider effects [30]. Furthermore, it was shown that although the overall *average error* in estimation of provider effects is smaller because mean square error is minimized over the full set of provider effects in the RE approach, FE estimates have smaller error for extreme “providers whose effects are exceptionally large or small” [30], which are the providers that a profiling analysis aims to identify. Our previous works also have shown that the benefit of stabilization via shrinkage comes at a severe cost of substantially biased provider effects estimation and, more importantly, a substantial reduction in the power to identify extreme providers [31, 33].

In the ESKD population, patient outcomes are not sparse, including mortality, hospitalization, and readmission which are very high [32–37]. (See Introduction Section for details.) In this context when the outcome is not sparse, the benefit from estimation shrinkage using a RE model is minimal and the diminished ability to flag extreme providers (e.g., to identify under-performing dialysis facilities) is a substantial disadvantage, especially when CMS payment is tied to providers’ performance status. These observations, combined with the aforementioned inherent estimation bias due to confounding between patient risk factors and provider effects for the RE model motivated researchers at the University of Michigan Kidney Epidemiology and Cost Center (UM-KECC) to propose high-dimensional FE models for profiling applications in the ESKD patient population [38]. Specifically, high-dimensional FEs models have been reported to be effective in flagging extreme dialysis facilities [38] while at the same time avoiding confounding between patient risk factors and facility effects [24, 30].

The high-dimensional FE profiling model proposed by He *et al*. (2013) [38] for hospital readmission, i.e., $Y_{ij}=1$ if the jth patient index hospitalization results in a readmission (and 0 otherwise), is

(2) $$g\left(\mu_{ij}\right)=\gamma_{i}+Z_{ij}^{T}\beta ,i=1,\ldots ,I,$$

where $\gamma_{1},\ldots ,\gamma_{I}$ are fixed facility (provider) effects to be estimated. We emphasize that the FE model (2) is a single simultaneous model for all I facilities. Thus, the model is high-dimensional because, in practice, profiling dialysis facilities involve over I=6,000 facilities and about 30 case-mix parameters β which require simultaneous estimation. (We note that FE model (2) is not to be confused with a single logistic regression with an overall intercept term which was also referred to as a “fixed effects model” in early literature on profiling; hence, we emphasize the descriptor “high-dimensional” FE for model (2) due to the high-dimensional parameter space $\left\{\gamma_{1},\ldots ,\gamma_{I},\beta_{1},\ldots ,\beta_{r}\right\}$.) With the high dimensionality, standard software cannot be used to fit model (2). Instead, an alternating one-step Newton-Raphson algorithm proposed by He *et al*. (2013) [38] can be used. This key FE methodology development period is represented in Figure 1 (bottom panel - blue) and parallels the publication/release of the COPSS white paper in 2012 [24].

Under profiling model (2) or (1), a performance (quality) measure is then defined to assess the performance of each provider with respect to a patient outcome. For example, consider the patient outcome of 30-day (unplanned) hospital readmission among hospitals (providers) in the US. Here, $Y_{ij}=1$ if the patient index discharge j at hospital i results in a readmission within 30 days (0 otherwise). For this outcome, model (2) is $\left(p_{ij}\right)=\text{log}\left\{p_{ij}\left(1-p_{ij}\right)\right\}=\gamma_{i}+Z_{ij}^{T}\beta$, where $\mu_{ij}=p_{ij}=\text{Pr}\left(Y_{ij}=1|Z_{ij}\right)$. A performance measure adjusted for patient case-mix commonly used is the standardize readmission ratio (SRR), defined as

(3) $$SRR_{i}=\frac{\sum_{j=1}^{N_{i}}\mu_{ij}}{\sum_{j=1}^{N_{i}}\mu_{ij,M}},$$

where $\mu_{ij}=g^{-1}\left(\gamma_{i}+Z_{ij}^{T}\beta \right)$ and in the denominator $\mu_{ij,M}=g^{-1}\left(\gamma_{M}+Z_{ij}^{T}\beta \right)$ with $\gamma_{M}$ denoting the median of the $\gamma_{i}$’s (i=1,…,F). (The median is used here instead of the mean as a robust measure of the “average” provider effect [38]). For the RE model (1), this quantity would be replaced by $\gamma_{0}$, since $\gamma_{i}\sim N\left(\gamma_{0},\sigma^{2}\right)$. An estimate of $SRR_{i}$ is then obtained by plugging in the model-based estimates ${\overset{ˆ}{\gamma }}_{i}$ and $\overset{ˆ}{\beta }$ from model (2). Note that when the patient outcome is (e.g., 30-day or in-hospital) mortality, the performance measure has the same form as (3) and is called standardized mortality ratio $\left(SMR_{i}\right.$ for provider $\left.i\right)$.

The numerator in $SRR_{i}$ in (3) is the (expected) total number of readmissions for provider i. The denominator is the expected total number of readmissions for an “average” provider (taken over the population of all providers), adjusted for the specific case-mix of the *same* patients in provider i. One can also interpret the denominator as a counterfactual: If the same patients receiving care at provider i were to receive care at a “national average provider,” then the expected total readmission is the denominator in $SRR_{i}$. Thus, $SRR_{i}=1\left(\gamma_{i}=\gamma_{M}\right),SRR_{i}>1$, and $SRR_{i}<1$ indicates that the readmission rate for hospital i is not different from the national norm (average over all hospitals in the US), greater than the national norm, and less than the national norm, respectively. Note that $SRR_{i}$ is an indirect standardization ratio [24, 46] and it is clear from (3) that one should not use this measure to compare among hospitals, i.e., comparing hospital A versus B by comparing $SRR_{A}$ and $SRR_{B}$. Doing so is generally not valid unless the patient case-mix characteristics of hospital A and B are the same. (See [24] for a more extensive discussion.) Indeed, CMS Hospital Compare reports whether a specific hospital is greater than, less than or not different than the national norm and provides the national norm/rate for specific patient outcome (e.g., readmission or mortality for all-cause or condition-specific) [29].

In addition to the novel FE model (3) and effective estimation procedure, researchers from the UM-KECC continue to advance FE modeling methodologies, including: 1) profiling hospital readmission accounting for competing risk [39]; 2) improved computational methods (e.g., serial blockwise Newton algorithm and shared-memory divide-and-conquer) for estimation and inference in FE profiling models [40]; 3) accounting for additional variation between facilities that are outside the facilities’ control using smoothed empirical null [41]; and 4) profile inter-unit reliability metric to monitor providers [42–44]. See Figure 1 (bottom panel).

Our own works also focus on profiling dialysis facilities for ESKD patient outcomes (Figure 1 – red box). More specifically, to address the need for longer-term monitoring of dialysis facilities, we proposed time-dynamic profiling of longitudinal patient outcomes, such as hospitalizations over time [35, 36]. For the vast majority of ESKD patients, dialysis is a life-sustaining treatment for the duration of their lives, and for about 30% of patients dialysis is a necessary treatment until kidney transplantation. Thus, *longitudinal metrics* can contribute to the overall performance assessment for dialysis providers. A second area of our works involve understanding dialysis facility factors that contribute to unplanned hospital readmissions of ESKD patients [37]. Because patients primarily receive dialysis treatment and care at dialysis facilities and they interact with nephrologists, nurses, PCTs, dietitians, and other dialysis facility staff as part of their care, adequate staffing as well as staffing composition may play a role in the rates of hospitalization and readmission. A third area involves development of FE profiling model and associated performance measure to assess recurrent adverse events (RAEs) related to dialysis [34]. Management/prevention of RAEs involves monitoring patient parameters, including phosphorous level, bone and mineral disorder, hemoglobin levels among others to avoid acute events that can lead to hospitalizations. Finally, a fourth area of interest is understanding the operating characteristics of FE profiling models, including its limitations. For instance, “How does it perform under (highly) sparse outcome data in terms of estimation and ability to flag extreme facilities?” [45] or the impact of inadequate case-mix adjustment [31]. In the subsequent sections of this paper, we review each of these four areas with a focus on the rationale of the modeling especially aspects relevant to the ESKD population, interpretation of results, and challenges for future research.

## 3. TIME-DYNAMIC PROFILING

For government agencies to identify providers (such as hospitals and dialysis facilities) with below standard performance (e.g., below a national average standard/norm) with respect to a regulatory objective, it is convenient and relatively straight forward to build a profiling model for a static (non time-varying) patient outcome. In this setting, profiling model (1) or (2) can be implemented annually for a specific policy objective, e.g., CMS reimbursement policy for hospitals. Annual/static profiling of providers are appropriate and useful particularly for the general population with respect to specific patient outcomes, including all-cause, cause-specific, or condition-specific (e.g., heart failure, chronic obstructive pulmonary disease [COPD] pneumonia) unplanned readmission. For many of these medical conditions, receipt of in-patient care (treatment) at a hospital is short-term so “continuous” care/monitoring spanning years is not applicable; therefore, annual profiling to assess providers’ performances is more useful.

However, for patients with ESKD, a unique population which requires continuous medical care, methodologies to monitor *longitudinal patient outcomes* over time provide distinct information on performance aspects of dialysis facilities that are not available through static profiling metrics. Secondly, although targeting a specific policy objective through payment/reimbursement based solely on static (short-term) quality measures can drive specific outcomes, this can also lead to other unanticipated systemic consequences. For instance, do short-term (e.g., annual) quality measures discourage providers from adopting longer-term innovations to achieve better patient outcomes over a longer time period? (This may include infection control monitoring protocol, care coordination between hospital and dialysis facilities, or incentivization structure for dialysis facility care teams that emphasizes continuous quality care and more regular/frequent follow up with dialysis patients.) A third important motivation for longitudinal quality metrics relates to the profiling objective of providing feedback to providers for quality improvement. For example, a time-dynamic measure that provides information on hospitalization or readmission rate over time starting from when patients transition to dialysis is useful to identify specific time periods of higher than expected hospitalization rates. Such information would be useful for providers to further examine care processes/factors for improvement in the flagged time periods of poor performance. These motivations underpin the development of time-dynamic profiling (TDP) methodology [35, 36].

### 3.1. Multilevel Varying Coefficient Model for TDP

TDP was proposed for multilevel data structure with patients nested (clustered) within facilities and repeated measurements (hospitalizations) over time for each patient. More formally, assume that we have a cohort of incident ESKD patients followed over time starting from their transition to dialysis. As before, i=1,…,I denotes dialysis facilities and $j=1,\ldots ,N_{i}$ denotes patients receiving dialysis treatment at facility i with $N_{i}$ total number of patients in facility i. An additional notation is needed to for repeated hospitalizations for each patient, therefore, let $k=1,\ldots ,N_{ij}$ index hospitalizations for patient j at facility i with $N_{ij}$ denoting the total number of hospitalizations for patient i during the patient’s follow-up time period. For longitudinal 30-day readmission, we denote the outcome variable as $Y_{ijk}\equiv Y_{ij}\left(t_{ijk}\right)\;$ which equals 1 if the k th index hospitalization for patient j results in a readmission within 30 days, and 0 otherwise. For this purpose, the following generalized (logistic) multilevel varying coefficient model (MVCM) was proposed [35],

(4) $$g\left\{E\left[Y_{ij}\left(t\right)|Z_{ij},b_{ij},t<S_{ij}\right]\right\}\equiv g\left\{p_{ij}\left(t\right)\right\}=\gamma_{i}\left(t\right)+b_{ij}+Z_{ij}^{T}\beta ,i=\cdots ,I,$$

where the fixed time-varying dialysis effect of facility i is captured by the varying coefficient function $\gamma_{i}(t),t$ is the time after transition to dialysis, $S_{ij}$ is the death time of patient $j,\;b_{i}={\left(b_{i1},\ldots ,b_{iN_{i}}\right)}^{T}$ is the subject-specific REs within facility i to account for within-subject correlation, and it is assumed that $b_{ij}\sim N\left(0,\sigma_{b}^{2}\right)$.

In model (4), $\;p_{ij}(t)\equiv E\left\{Y_{ij}(t)|Z_{ii},b_{ii},t<S_{ii}\right\}=g^{-1}\left\{\gamma_{i}(t)+b_{ij}+Z_{ij}^{T}\beta \right\}$ denotes the “partly conditional” target of inference, conditional on patients being alive: $t<S_{ij}$. Partly conditional target of inference was previously considered in [47] as well as in modeling cardiovascular hospitalizations in the ESKD patients [48, 49]. Note that the generalized MVCM (4) extends the FE logistic regression model (2) for time-static dialysis facility profiling, to model time-dynamic facility effects via facility-level varying coefficient functions, which are the main goal in TDP. Also, similar to model (2), the set of high-dimensional parameters $\left\{\gamma_{1}(t),\ldots ,\gamma_{I}(t),\beta_{1},\ldots ,\;\beta_{r},\sigma_{b}^{2}\right\}$ requires simultaneous estimation. For details of the estimation procedure the reader is referred to [35].

Under the MVCM model (4), a measure to assess the time-dynamic performance for facility i relative to a (time varying) reference norm, accounting for patient case-mix, is the standardized dynamic readmission ratio (SDRR),

(5) $${\text{SDRR}}_{i}\left(t\right)=\frac{\underset{j\in {\mathbb{N}}_{it}}{\sum }p_{ij}\left(t\right)}{\underset{j\in {\mathbb{N}}_{it}}{\sum }p_{ij,M}\left(t\right)}$$

where the summation is taken over the set of all patients in facility i and alive at time t, denoted by $N_{it}$. In the denominator, $p_{ij,M}(t)=g^{-1}\left\{\gamma_{M}(t)+b_{ij}+Z_{ij}^{T}\beta \right\}$, where $\gamma_{M}(t)$ is the cross-sectional median of $\left\{\gamma_{1}(t),\ldots ,\gamma_{I}(t)\right\}$. The time varying function ${\text{SDRR}}_{i}(t)$ is the ratio of the total expected number of readmissions for all patients alive at facility i at time t relative to the total expected number of readmissions for the same patients at time t based on the reference norm (e.g., national average rate at time t). An appealing aspect of (5) is that it provides a natural graphical summary of the time periods of under-performance and over-performance, and time periods where readmission rates are not different from the reference norm, corresponding to $SDRR_{i}(t)$ significantly greater than 1, significantly less than 1, and not significantly different from 1, respectively.

### 3.2. Application to Longitudinal Hospitalization Trajectories in ESKD Patients

TDP model (4) was applied to assess the performance of dialysis facilities with respect to 30-day hospital readmission for patients 18 years of age or older with ESKD who transitioned to dialysis between January 1, 2006 to December 31, 2009 using data from the USRDS [35]. The USRDS is a national registry that includes nearly all patients with ESKD in the US. This included 113,764 patients receiving dialysis care at 2,896 facilities. A critical component of profiling is the risk adjustment. For this, adjustment for patient factors similar to CMS model for 1-year profiling [53] were made, including (1) 23 previous year comorbidities determined by claims data in the 12 months prior to the initiation of dialysis treatment for each person; (2) indicator of a high-risk hospitalization during the year prior to the start of dialysis; and (3) other patient-level factors adjusted for included age, sex, body mass index (BMI: underweight, normal, overweight, obese), and diabetes as the cause of ESKD. Note that this set of risk-adjustment factors capture the baseline characteristics of the patients, including comorbidities, prior to the start of the longitudinal follow-up (start of dialysis treatment).

Table 1 summarizes the main comorbidities significantly associated with longitudinal 30-day hospital readmission from fitting model (4). Risk factors ascertained through claims data in the previous 12 months significantly associated with higher odds of 30-day readmission include drug and alcohol disorders (odds ratio [OR] 1.37); end-stage liver disease (OR 1.22); metastatic cancer/acute leukemia (OR 1.27); severe hematological disorders (OR 1.17); pancreatic disease (OR 1.26); severe cancer (OR 1.16); transplants (OR 1.10); seizure (OR 1.21); and high risk hospitalization (OR 1.52).

**Figure 2:**
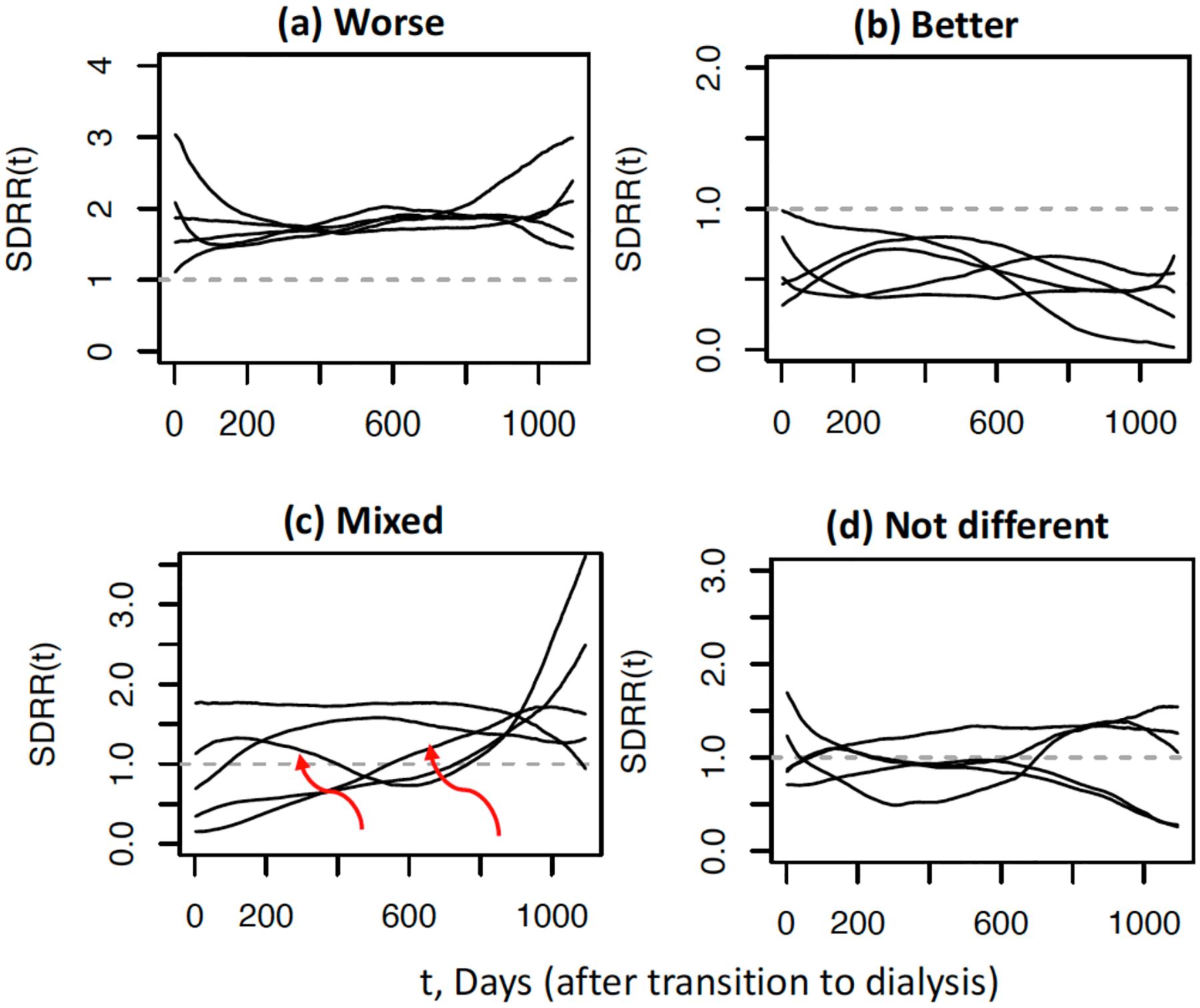
Estimated standardized dynamic readmission ratio (SDRR(t)) as a function of time t (days since transition to dialysis); displayed are five dialysis facilities found to have readmission rates significantly (**a**) worse, (**b**) better, and (**d**) not different relative to the national norm. Shown in (**c**) are five dialysis facilities with SDRR(t) that significantly vary over time with some time periods worse, better, or not different (mixed) compared to the national norm. Adapted from [35].

Next, we illustrate the graphical display of dialysis facilities’ longitudinal performance with respect to 30-day readmission in Figure 2. Shown in Figure 2a are five distinct facilities found to have significantly higher readmission across time (over 3 years of follow-up from when patients transitioned to dialysis) relative to the national norm (average rate) with ${\text{SDRR}}_{i}(t)>1$, for all time t. These facilities have rates about 1.5 to 1.7 times higher than the national norm between 6 months and 1.6 years. Furthermore, 4 of the 5 facilities displayed have rates steadily increasing after 1.6 years. Such facilities can be flagged for examination of factors that contributed to higher than expected rates of (unplanned) 30-day hospital readmission. Similarly, five distinct facilities with significantly better than expected rate of 30-day readmission are displayed in Figure 2b where $SDRR_{i}(t)<1$ for all time t. Figure 2d illustrate five selected dialysis facilities whose 30-day readmission rates are not significantly different then the national norm; i.e., ${SDRR}_{i}(t)$’s are not significantly different from 1. It is also possible that for some facilities, readmission rates vary over time with some time periods of worse, better, or not different (mixed) performance compared to the national norm; i.e., mixed pattern of $SDRR_{i}(t)$. This is illustrated in Figure 2c with five distinct facilities, where two facilities are highlighted (red arrows): (1) One facility had readmission rate over time significantly better than the national norm until about 1.5 years where rate begins to increase and becomes significantly worse than expected and (2) a second facility with $SDRR_{i}(t)$ varying about 1 (not different from the national norm), but rises rapidly after 1.92 years (~ day 700). Again, similar to facilities with significantly worse than expected performance, these facilities with (significantly) mixed patterns of readmission rates can be further studied to determine potential factors or changes in factors at the facilities that may have contributed to significant variation in readmission rates over time.

Since our main goal of this review paper is on the modeling, rationale, and applications we refer the interested readers to [35] for details on estimation and inference procedures. However, we note here the main ideas. For model estimation, let $L_{ij}\left\{\gamma_{i}(t),\beta \right\}$ denote the joint distribution of the response $\left\{Y_{ij1},\ldots ,Y_{ijN_{ij}}\right\}$ at time $t_{ij}=\left(t_{ij1},\ldots ,t_{ijN_{ij}}\right)$, where $t_{ij}<S_{ij}$ and for simplicity consider model (4) without subject-specific random effects. The model likelihood is $L\left\{\gamma_{1}(t),\ldots ,\gamma_{I}(t),\beta \right\}=\prod_{i=1}^{I}\;\prod_{j=1}^{N_{i}}\;L_{ij}\left\{\gamma_{i}(t),\beta \right\}$ which is separable into I components, where the ith component, specifically $\prod_{j=1}^{N_{i}}\;L_{ij}\left\{\gamma_{i}(t),\beta \right\}$, depends only on $\left\{\gamma_{i}(t),\beta \right\}$. Due to this factorization, given $\beta ,\gamma_{i}(t)$ can be estimated by maximizing the local likelihood based on data only from the ith facility; and given the facility effects $\left\{\gamma_{1}(t),\ldots ,\gamma_{I}(t)\right\},\;\beta$ can then be estimated based on a global likelihood without the need for localization. Thus, an iterative Newton-Raphson algorithm can be implemented together with an approximate EM algorithm. For model inference, specifically for identification of extreme facilities (as illustrated in Figure 2) a hypothesis testing procedure can be used, where the null hypothesis is $H_{0}:{\text{SDRR}}_{i}(t)=1$ for all t. Because under the null, $\sum_{j\in N_{it}}\;p_{ij}(t)=\sum_{j\in N_{it}}\;p_{ij,M}(t)\;$ or $\gamma_{i}(t)=\gamma_{M}(t)$), the test statistic is defined as the departure of $\sum_{j\in N_{it}}\;{\overset{ˆ}{p}}_{ij}(t)$ from $\sum_{j\in N_{it}}\;{\overset{ˆ}{p}}_{ij,M}(t)$ using $L^{2}$-norm; specifically the test statistic is ${\left[\int {\left\{\sum_{j\in N_{it}}\;{\overset{ˆ}{p}}_{ij}(t)-\sum_{j\in N_{it}}\;{\overset{ˆ}{p}}_{ij,M}(t)\right\}}^{2}dt\right]}^{1/2}$. For a detailed description of the model estimation and hypothesis testing procedure, see Web Appendix A and B to [35], respectively.

### 3.3. Discussion, Extensions and Future Research

There are several extensions to the TDP approach. First, the TDP framework described above is based on a partly conditional target of inference, which characterizes the 30-day readmission outcome among dialysis facilities conditional on the patients being alive and does not include death as another joint outcome. Therefore, if patient care within facilities is associated with varying mortality, this information is not directly accounted for in the current TDP modeling. Thus, consideration of joint patient outcomes, such as 30-day hospital readmission and mortality, as an extension to the TDP is of interest. Bivariate time varying measures, extending $SDRR_{i}(t)$, for both hospitalization and mortality, can be developed for the joint outcomes. Our own works have recently considered joint modeling of hospitalization and survival in ESKD patients [50–52] in the standard joint modeling context. Extension of joint modeling for TDP would require methodological developments as well as innovative computational approaches to overcome the challenges in estimation and inference for a high-dimensional parameter space in the joint outcomes profiling setting.

In a second approach, with the inferential focus still on time-dynamic hospitalization, one can treat death as a competing risk. In the static profiling setting, profiling model accounting for competing risk has been explored in [39].

A third area of research, which encompasses the TDP described above as well as extension to joint modeling and competing risk, is to improve the overall model structure for dialysis facility effects. Currently, for static profiling as well as TDP, it is assumed that once patient risk factors/case-mix have been thoroughly accounted for (i.e., through adjustment for $Z_{ij}$) remaining outcome variation is due to dialysis (provider) effects (i.e., process of care). Improvement to this decomposition of patient outcome variation is of practical interest. For example, in the context of profiling hospitalization for dialysis facilities, this includes factors that are unrelated to dialysis facilities (e.g., hospital effects [38]) or even “process of care factors” unrelated (not attributable) to dialysis facilities. This would need to be considered carefully and involving all relevant stakeholders. For a more detailed discussion see [36].

## 4. IDENTIFYING DIALYSIS FACILITY CHARACTERISTICS ASSOCIATED WITH HIGH RATES OF UNPLANNED HOSPITAL READMISSION

As introduced in earlier sections, further understanding of factors that contribute to patient outcomes, including those that are directly process of care factors, structural resource factors, or even incentive structures within providers, is important for quality improvement. As summarized above, unplanned hospital readmissions are a major source of morbidity among ESKD patients on dialysis for whom the risk of hospital readmission is exceptionally high. In this section we illustrate an approach to examine dialysis facility characteristics associated with 30-day (unplanned) hospital readmission among patients with ESKD [37], such as patient-to-staffing ratios and staffing composition (e.g., nurses-to-total staff). The contribution of dialysis facility staffing to hospital readmission is an area that has been largely overlooked.

### 4.1. Two-Stage Exploratory Analysis to Identify Facility Risk Factors

Before describing a two-stage exploratory analysis for this purpose, we first return to the important issue of adequate patient case-mix adjustment described in Section 2. For any profiling model it is critical to include all relevant *patient risk factors* $\left(Z_{ij}\right)$ so that it is reasonable to assume that the remaining variation in patient outcomes after risk adjustment can be attributable to facilities. First, these risk factors should typically be related to patients’ health condition, such as comorbidities at baseline. Furthermore, depending on the overall policy objective, certain patient factors (e.g., race, socioeconomic, education, or some “social determinants of health”) may not be appropriate for risk adjustment, if, for instance, the policy objective is to eliminate disparity in health outcomes across race categories and socioeconomic status. Second, the risk adjustment should not include factors related to the treatment “process”. Note that “treatment-related” can be viewed in the broadest terms, including institutional structures and processes (e.g., hospital allocation of resources to departments, management processes, staffing, infection control protocol, discharge policy and coordination etc.). Furthermore, this should not include time-varying factors possibly related to facility effects. We note that a common misunderstanding in profiling analysis centers on the intuition to include (adjust for) factors related to the process of care, such as the effect of nephrologist or nephrology care or other facility-level factors including management strategies (e.g., infection-control policy). Unless there is justification underlying an explicit policy objective, inclusion of such factors is generally not appropriate. Generally, no facility-level factors or no factors on the “causal pathway” after the start of follow-up should be included as a basis to assess SRR [24, 32, 38] because doing so would explain away variation in outcomes presumably attributable to dialysis facilities. This risk adjustment step is critical in stage 1 of the analysis.

**Figure 3:**
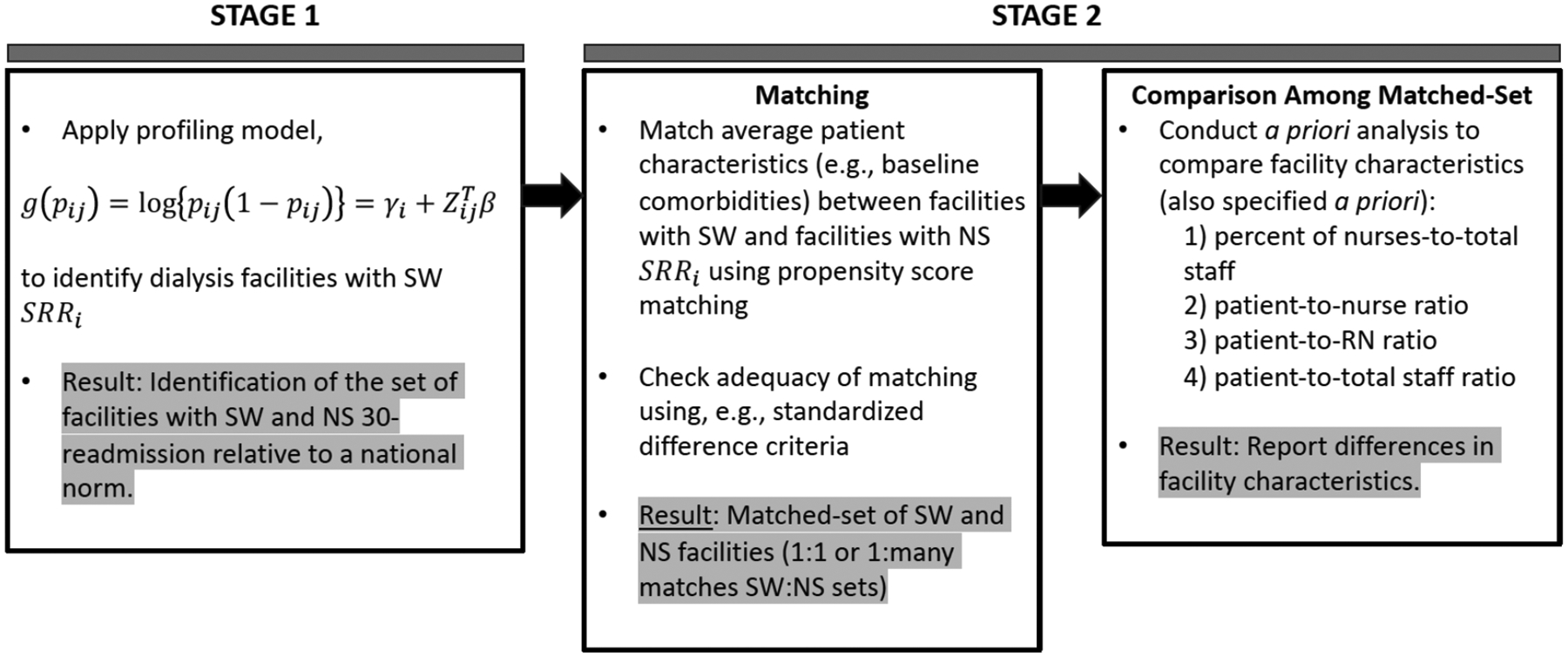
Outline of two-stage analysis: (1) profiling modeling and (2) creating matched-sets of facilities of significantly worse (SW) standardized readmission ratio (SRR) and facilities with SRR not significantly (NS) different relative to the national norm/average rate for comparative analysis of facility characteristics.

A two-stage analysis can be used to avoid confounding of the patient-care process variables (facility staffing) with patient-risk factors and the outcome, 30-day (unplanned) readmissions. In the first stage, profiling model (2) or (1) can be used to estimate facility effects $\gamma_{i}$’s (the contribution of a facility total process of care towards hospital readmission of patients at that facility). In the first stage analysis, facilities with 30-day readmission rates that are significantly worse (SW) than expected relative to the national norm are identified (i.e., with $SRR_{i}>1$ significantly). These are identified using a hypothesis testing procedure that test the null hypotheses $H_{0}:\gamma_{i}=\gamma_{M}$ (i.e., $SRR_{i}=1$) for FE model (2) [38] or via a bootstrap confidence interval (CI) for RE model (1) [16, 24]. Details are provided in the Appendix section. In stage 2, a comparison of facility staffing variables between facilities with SW readmission and facilities with readmission rates not significantly (NS) different from the average rate can be performed. However, a direct comparison of patient-care staffing variables between SW and NS facilities may not be appropriate generally because there may be differences in patient case-mix characteristics, including baseline comorbidities, between flagged SW facilities relative to NS facilities. Thus, in stage 2, analysis to match facilities with NS readmissions to facilities with SW readmissions with respect to average patient risk factors as well as facility size (number of patients) is needed. Because of the large number of patient risk factors, matching can be based on the propensity score [54, 55] and adequacy of matching can be assessed by checking the balances of covariates before and after matching using the absolute standardized difference criteria [56]. Finally, since patient staffing variables are continuous variables, comparisons between matched-sets of SW and NS facilities can simply be based on multiple linear regression models (to doubly adjust for average patient risk factors) or t-tests comparisons. These analysis models should be specified *a priori*. Figure 3 summarizes the two-stage exploratory analysis. An illustrative analysis is provided in the Section 4.2 next.

### 4.2. Application and Results

In stage 1, we applied profiling models (2) and (1) for ESKD patients’ readmission data for each year from the year 2010 to 2013 which included a range of about 135.8K (thousands) to 148.1K discharges per year and over 5,000 facilities in each year [37]. Similar to CMS profiling models for 30-day readmission, we risk adjusted for age at hospitalization, sex, BMI, diabetes as the cause of ESKD, years on dialysis, length of index hospitalization, high-risk index hospitalization, and 23 past-year comorbidities [37]. The overall rate of dialysis facilities flagged as having SW performance by at least one model was similar for 2010 to 2013: 4.2% (222/5346), 3.8% (211/5579), 3.1% (176/5637) and 3.0% (171/5628).

**Figure 4:**
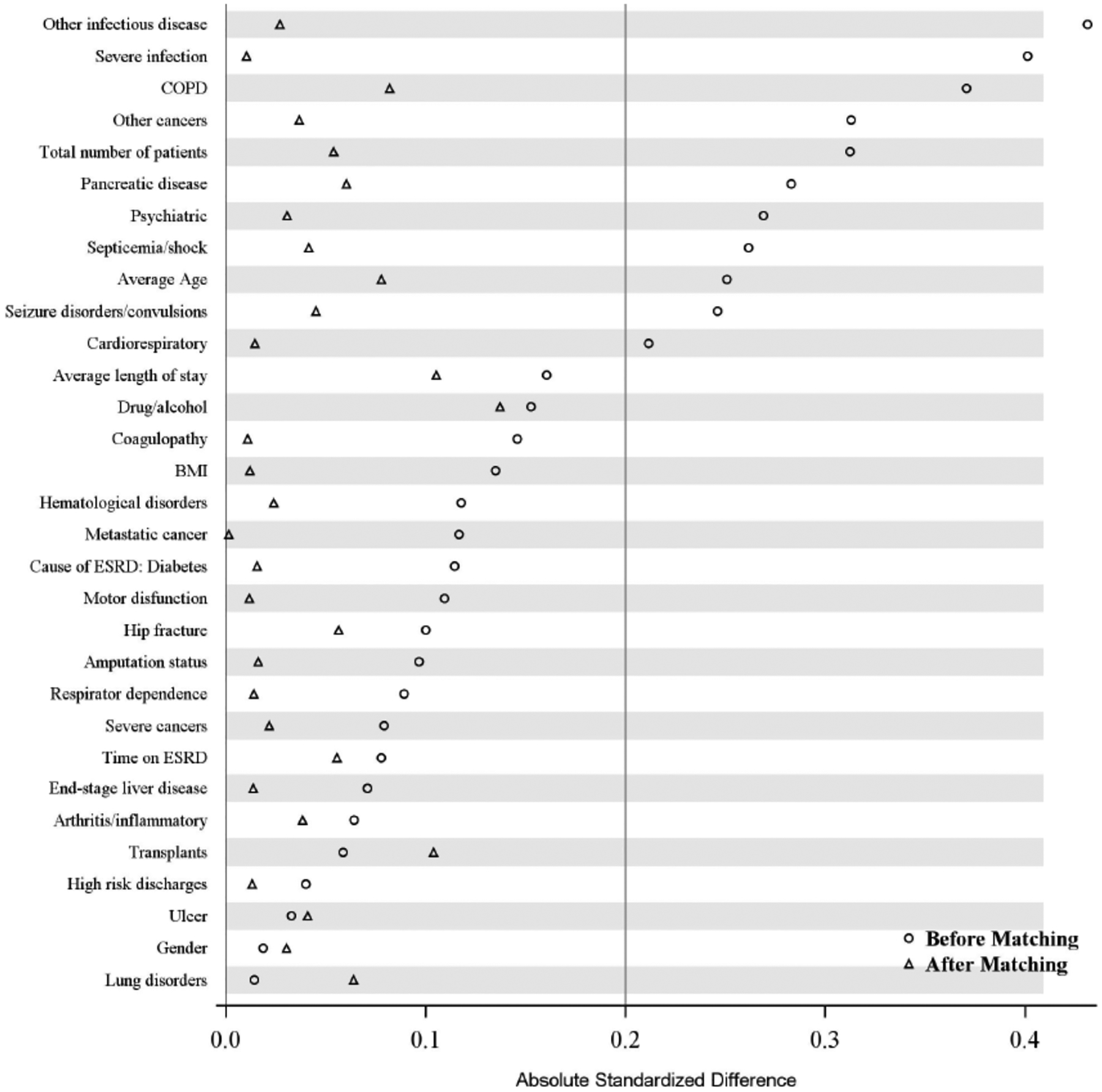
Patient demographic, risk factors and facility size before and after propensity score matching. Shown are results for the year 2012.

Because the average patient characteristics between SW and NS facilities were found to be different (Figure 4 – Before matching), propensity score matching was used to find matched sets of SW and NS facilities so that on average there were no substantive difference between groups (SW vs. NS) with respect patient characteristics, including comorbidities and facility size. Figure 4 illustrates the success of propensity score matching to balance average patient covariates between SW and NS facilities. (All absolute standardized differences were less than 0.2 after matching).

**Figure 5:**
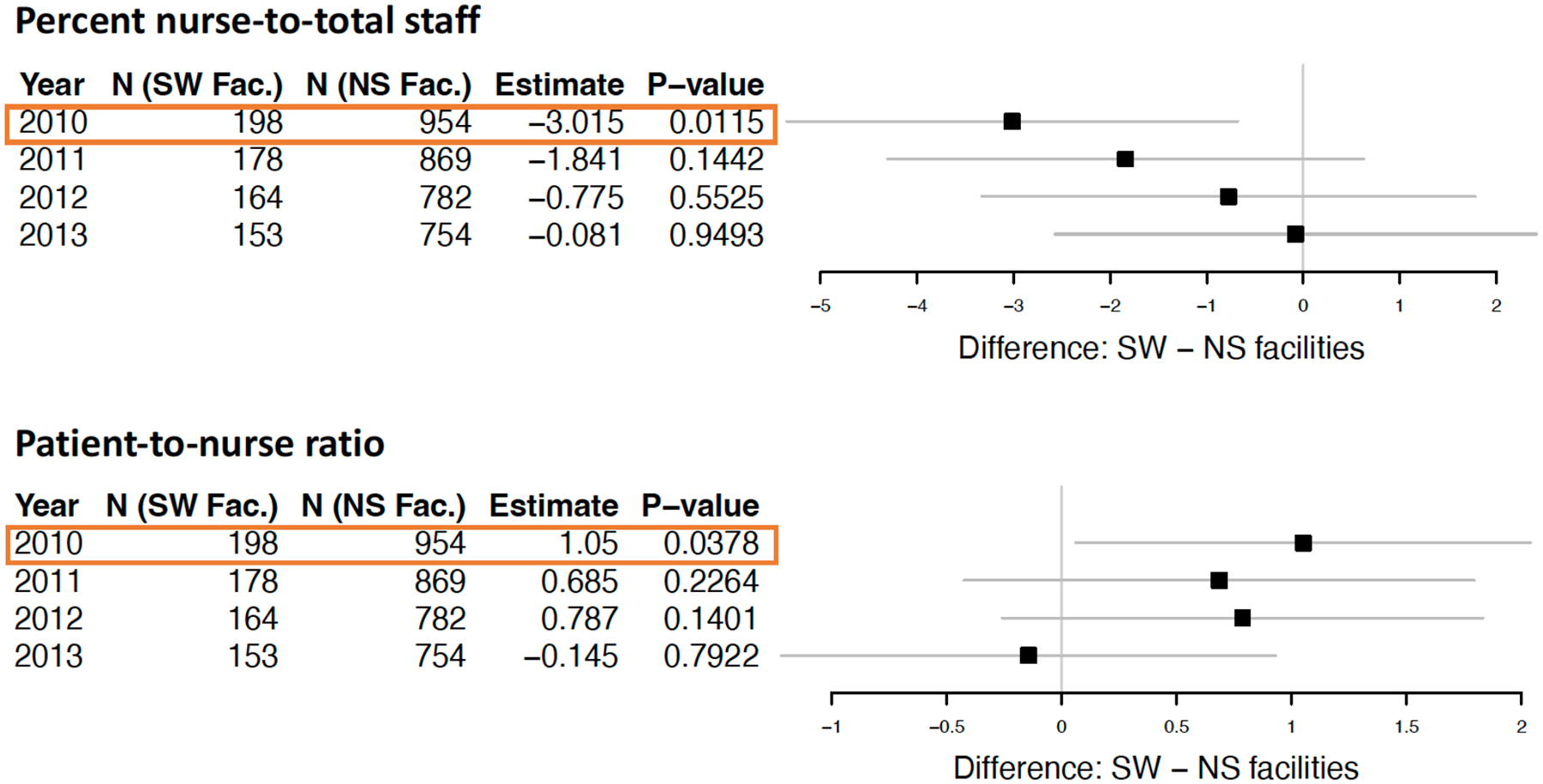
Differences in percent nurse-to-patient staff and patient-to-nurse ratios in dialysis facilities with significant worse than expected 30-day readmission compared to facilities with 30-readmissions not different relative to the national norm. Adapted from [37].

Using the matched sets for each year, comparison between SW and NS facilities were carried out with respect to four patient-care staffing outcomes in stage 2 analysis: 1) percent of nurses-to-total staff, 2) patient-to-nurse ratio, 3) patient-to-RN ratio, and 4) patient-to-total staff ratio. The percent of nurses-to-total staff was significantly lower in 2010 for SW facilities compared to matched NS facilities (42.5% vs. 45.6%, p = 0.012), but this disparity was attenuated by 2013 (44.8% vs. 44.7%, p = 0.949). There was higher patient-to-nurse ratio for SW facilities compared to NS facilities (mean 16.4 vs. 15.2, p = 0.038) in 2010 as well, and the disparity was reduced by 2013 (see Figure 5). The trends were similar for patient-to-total staff and patient-to-registered nurse, but not statistically significant (results not shown).

### 4.3. Discussion, Extensions and Future Research

As discussed in [37], staffing levels and composition in dialysis facilities are ultimately modifiable factors that can be optimized and potentially improve patient outcomes [57, 58]. Indeed, previous studies have shown that structural staffing issues, specifically inadequate nurse staffing in acute care hospitals have been linked to heightened risk of infection [59], mortality [60] and other adverse consequences [61–64]. As discussed in [65, 66], adequacy of dialysis clinic staffing and quality of care are linked; however, what constitutes “adequate” staffing has not yet been defined and requires further research and particularly with a focus on quality improvement.

Another area that deserves further examination is the level of coordination of care between hospitals and dialysis facilities after patient discharge. This is important to prevent unnecessary readmissions since the dialysis facility patient-care staff may not see patients post discharge until the next dialysis session which could be several days later. Furthermore, as discussed in [67] important key clinical parameters (e.g., anemia, serum albumin, and mineral metabolism and bone disease) as well as dry weights may have substantially changed over the course of hospitalization, and in prior studies of dialysis patients clinical parameters were found to be significantly changed after hospitalization [67]. Therefore, close care coordination and discharge planning are needed to potentially prevent recurrent illness in the days immediately following discharge that may contribute to hospital readmissions. Studies to compare care coordination, as well as other factors like infection control protocols and cardiovascular disease management protocols between SW and NS dialysis facilities would be of interest since the two most common causes of hospitalizations in ESKD patients are due to infections and CV disease.

## 5. PROFILING RECURRENT ADVERSE EVENTS IN ESKD PATIENTS

Next, we consider a third area of FE profiling application with respect to recurrent adverse events in ESKD patients. Patients with ESKD typically dialyzes 2–3 times per week, are monitored regularly, and have regular interactions with their dialysis care team, including nephrologist, nurses, PCTs, dietitians, and other staff. For example, patients receiving dialysis care at over 6,000 dialysis facilities across the US typically dialyze three times per week and they are monitored regularly with respect to a variety of patient outcomes, including dialysis adequacy (sufficient removal of waste from blood); bone and mineral disorder (e.g., to prevent high calcium in the blood or hypercalcemia); phosphorous level; regulation of blood pressure; and hemoglobin (Hb) to manage anemia among other conditions. Management of anemia, for instance, contributes to improved cardiovascular health, reduced risk of hospitalization, and prevention of fatigue [10]. Keeping these parameters within clinically acceptable ranges, i.e., avoiding these recurrent adverse events (RAEs), not only reduce the risk of hospitalizations, but also improve patients’ quality of life. Thus, monitoring dialysis facilities with respect to patients’ RAEs is an application of interest.

### 5.1. Method

To illustrate the FE profiling method for RAEs, we consider monitoring of Hb level. The outcome is the count of the number of times Hb levels are outside the target range (Hb between 9 and 11 g/dL) during the patient’s follow-up time period. Let $Y_{ij}$ be the number of RAEs for the jth patient during follow-up time $t_{ij}$. An appropriate profiling model for the count of RAEs (or equivalently the rate of RAEs: $Y_{ij}/t_{ij}$) is the log-linear profiling model [34],

(6) $$\text{log}\left\{E\left(Y_{ij}|Z_{ij},t_{ij}\right)\right\}\equiv \text{log}\left(\mu_{ij}\right)=\text{log}\left(t_{ij}\right)+\gamma_{i}+Z_{ij}^{T}\beta ,i=1,\ldots ,I,$$

Where $\mu_{ij}=\text{exp}\left\{\text{log}\left(t_{ij}\right)+\gamma_{i}+Z_{ij}^{T}\beta \right\},\;\gamma_{i}$ is the effect of dialysis facility i. We consider a Poisson model for the count outcome $\left(Y_{ij}\sim \text{Pois}\left(\mu_{ij}\right)\right)$ as well as a negative binomial model with mean $E\left(Y_{ij}\right)=\mu_{ij}$ and variance $Var\left(Y_{ij}\right)=\mu_{ij}\phi$, where ϕ is the overdispersion parameter, to directly account for potential overdispersion. To assess the performance of the facility i under model (6), the following risk adjusted measure, called standardized event ratio (SER) [34] can be used:

(7) $$SER_{i}=\frac{\sum_{j=1}^{N_{i}}\mu_{ij}}{\sum_{j=1}^{N_{i}}\mu_{ij,M}}.$$

In the denominator of (7), $\mu_{ij,M}=\text{exp}\left\{\text{log}\left(t_{ij}\right)+\right.\left.\gamma_{M}+Z_{ij}^{T}\beta \right\}$, where $\gamma_{M}$ is the median of $\left\{\gamma_{1},\ldots ,\gamma_{I}\right\}$, similar to standardized readmission ratio measure described earlier. The interpretation of SER parallels that of SRR: When $SER_{i}=1\left(\gamma_{i}=\gamma_{M}\right)$, the adverse event rate for facility i does not differ from the national norm, and when $SER_{i}>1$ or $SER_{i}<1$ then the event rate for facility i is greater or less than the national norm, respectively. Note that the SER measure (7) reduces to the SRR measure (3) when the outcome is binary (as in 30-day hospital readmission or mortality). The inference procedure (flagging extreme facilities) is outlined in the Appendix and details can be found in [34].

### 5.2. Application: Profiling Dialysis Facilities for Recurrent Anemia Events

We illustrate the log-linear profiling model (6) to assess the performance of dialysis facilities for recurrent anemia events using data on ESKD patients from the USRDS for the year 2014.

More specifically, the study cohort included prevalent and incident dialysis patients from January 1, 2014 through December 31, 2014 with Medicare as primary payer in the US. Patients were followed until kidney transplant, renal function recovery, death, or to the end of 2014 (82.6% of patients). Note that because patients on dialysis may switch facility where they receive dialysis treatment during the year (e.g., a patient’s residence may change), only the portion of their time spent at a specific facility was attributed to that facility accordingly. The final analysis cohort included 440,107 patients at 6,188 dialysis facilities where the number of patients per facility range from 10 to 560.

The patient outcome is the rate of adverse events, $Y_{ij}/t_{ij}$, where the count $Y_{ij}$ is the number of times a patient’s Hb level is outside the Hb target range and $t_{ij}$ is the total follow-up time for patient j in facility i (median follow up of 11 months). Recurrent anemic adverse events are common in ESKD patients where the mean rate is 5.6 times per year. For risk adjustment, patient risk factors included were similar to those described in the previous sections, except with two new risk factors of whether the patient: 1) had a Hb level out of the target range in the prior year and 2) received nephrology care prior to the patient’s transition to dialysis. (Note that for prior nephrology care, this is not attributable/related to the care process of the current (year 2014) facility in which patients received their dialysis.) We refer the reader to [34] for the effects of all the risk factors on the outcome and note that the largest effect size is whether patients had a RAE in the prior year (rate ratio: 1.302; 95% CI: 1.295–1.320).

**Figure 6:**
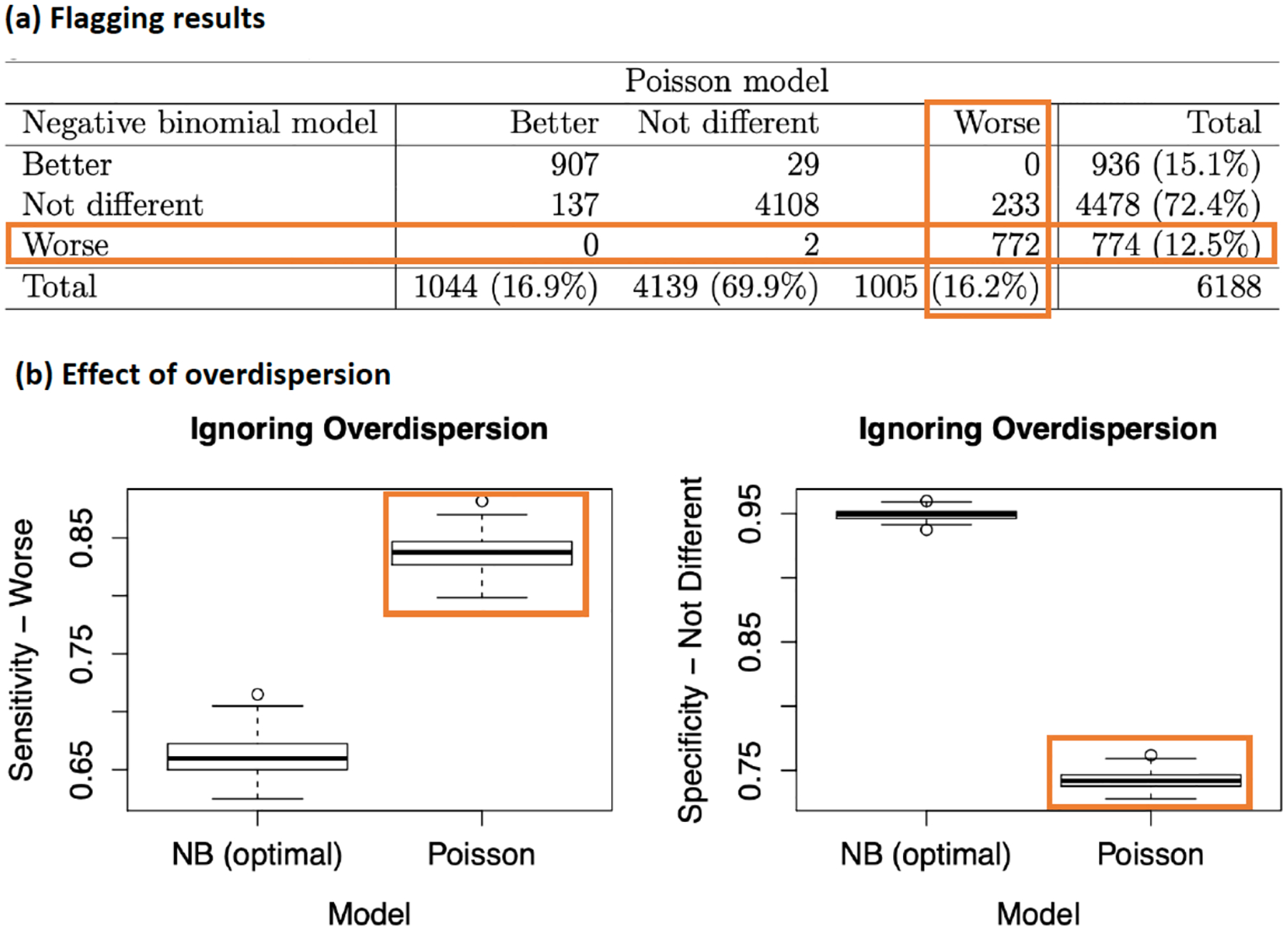
(**a**) Dialysis facilities flagged for recurrent adverse events (RAEs: anemic events) using Poisson and negative binomial models for RAEs outcome. (**b**) Impact of overdispersion on flagging facilities as significantly worse (sensitivity – worse) and not different (specificity) relative to a reference norm in simulation. Adapted from [34].

The results of flagging extreme facility performances using Poisson and negative binomial models for RAEs outcome (anemic events) in the log-linear profiling model (6) are summarized on Figure 6. Under the Poisson model and negative binomial model, 16.2% (1005) and 12.5% (772) of dialysis facilities were flagged as having significantly higher rate of anemic events than expected relative the national norm, respectively (Figure 6a). There was moderate overdispersion in the outcome with overdispersion parameter estimate of 1.4. The higher rate of worse performing facilities flagged under the Poisson model (which ignores overdispersion), compared to the negative binomial model, is consistent with simulation studies showing that ignoring overdispersion leads to over identification of extreme providers [34]. Figure 6b illustrates this phenomenon where the under a negative binomial model with overdispersion (optimal/true model), the Poisson model which does not incorporate overdispersion leads to inflated rate of worse providers (and reduced rate of facilities flagged as not different relative to the reference norm).

### 5.3. Discussion, Extensions and Future Research

Static annual profiling of RAEs for prevalent and incident patients during a given year, as illustrated for anemic events above, provide useful information for regulatory objectives as well as feedback to facilities for quality improvement. In addition to modeling counts of specific types of events, such as anemia, other events of interest include hypercalcemia, dialysis inadequacy, hyperphosphatemia etc., in the dialysis population. Furthermore, in practice, it may be informative to also consider combination of events deemed important for monitoring dialysis facilities. For example, the outcome of interest may be the number of times a patient experience multiple RAEs.

Also, to augment static profiling of RAEs, TDP of adverse events is also of interest because improved management of adverse events and symptoms over longer period of time for dialysis patients contribute to their overall quality of life as well as prevention of acute medical events such as hospitalizations. Finally, we note that multivariate profiling approaches that models multiple RAEs simultaneously/jointly have not been developed to date.

## 6. OPERATING CHARACTERISTICS OF FE METHOD

In this section we summarize several studies, including our own, that examined the performance of FE profiling method and comparisons to RE profiling approach.

### 6.1. Performance in Identifying/Flagging Underperforming Providers

As introduced in Section 2, a justification for the use of RE models is that they provide stable provider effect estimates through shrinkage [24] and account for the hierarchical data structure. This is indeed achieved with RE modeling, but at an important cost/disadvantage with respect to one of the main aims of profiling: to identify/flag under-performing providers. Using a simple regression model, the work of Kalbfleisch and Wolfe [30] succinctly illustrates the following findings.

- RE model estimates are biased toward the overall provider average and biased in the presence of confounding between patient risk factors and provider effects.
- Although the overall *average error* in estimation of provider effects is smaller for RE model, because mean square error is minimized over the full set of provider effects, FE estimates have smaller error for extreme “providers whose effects are exceptionally large or small,” which are the providers that a profiling analysis aims to identify.

Using the hierarchical logistic profiling model (1) and FE model (2) Chen *et al*. [31] compared the ability of the methods to flag extreme providers in simulation studies. The study utilized the same inference procedure used by CMS RE model to identify extreme providers (e.g., hospitals), which is a bootstrap resampling of providers with replacement (500 samples) and obtaining 95% CI for each SRR (or SMR). (See the Appendix section for details.) The simulation studies found the following.

**Figure 7:**
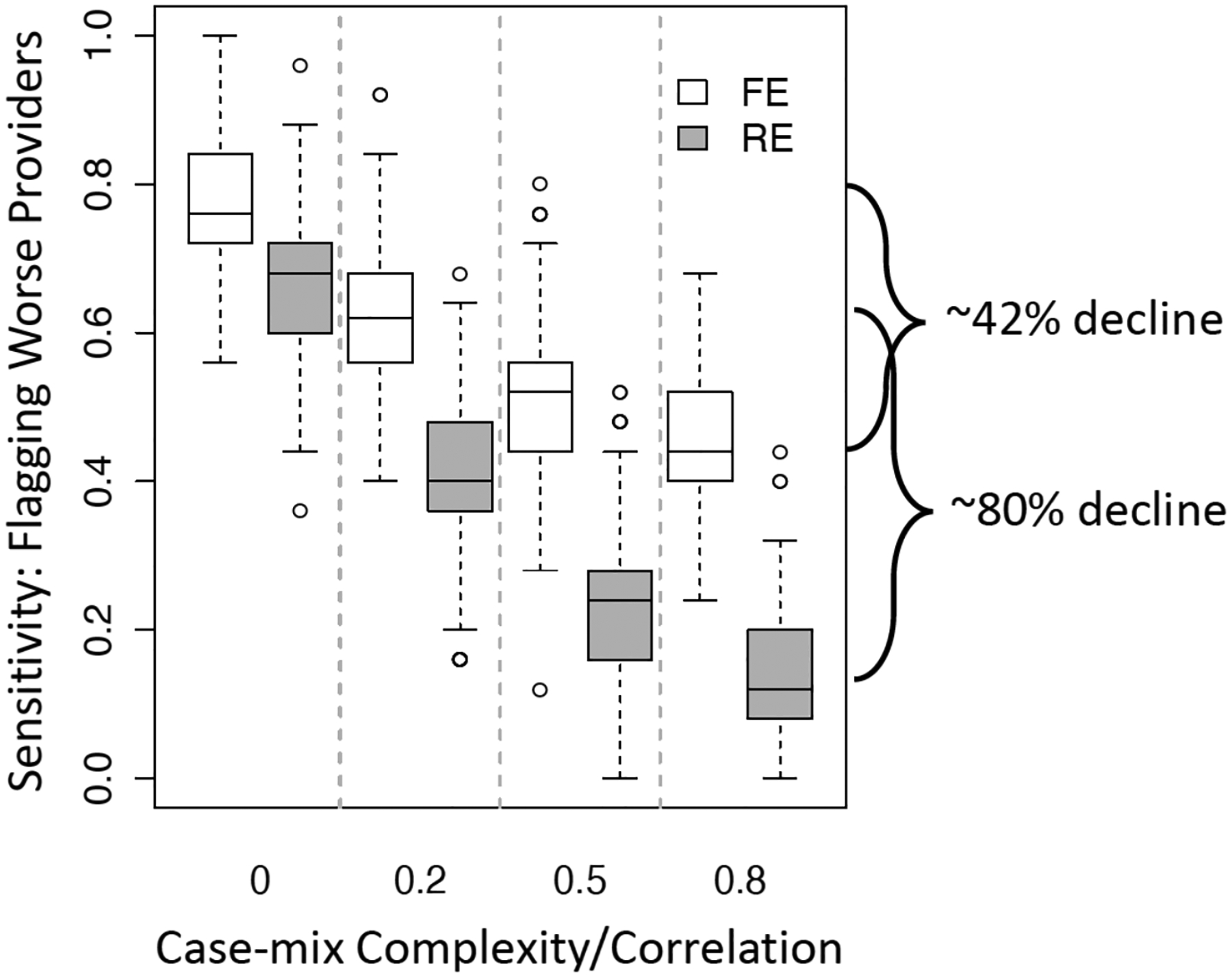
Sensitivity or rate of correctly flagging truly worse providers for fixed effects (FE) and random effects (RE) profiling model for increasing case-mix complexity (correlation among patient risk adjustment variables.) Adapted from [31].

- FE model outperformed RE model in identifying truly worse providers. (The study considered overall readmission rates of 14%−40% which are commensurate with various ESKD outcome prevalence rates.)
- When the case-mix complexity (defined as the correlation level among patient risk adjustment covariates) increases, the performance of both methods deteriorates, but RE performance deteriorates much more rapidly. An example of this is in Figure 7 (for 27% overall readmission rate) which illustrates that the rate of detecting truly worse providers declined by about 42% and 80% for FE and RE, respectively, when the correlation level among case-mix variables was high (~0.8) relative to uncorrelated case-mix covariates.
- This pattern of performance was similar when examined by provide volume (volume: small, medium, large).

### 6.2. Impact of Inadequate Case-mix Adjustment on Identifying Extreme Providers

A cornerstone of profiling analysis is the patient risk adjustment. Achieving adequate risk adjustment is important so that the modeling assumption that the remaining variation in patient outcome, after adequate patient risk adjustment, can be reasonably attributed to provider effects. The study in [31] supports this conclusion, and furthermore, found in simulation studies that the impact of inadequate case-mix adjustment (limited adjustment vs. optimal/full case-mix adjustment) on the sensitivity to identify truly worse providers was more than 30% for both FE and RE models on average. This reduction in performance is similar in size compared to the effect of model choice (i.e., choice of RE vs. FE model).

### 6.3. Case-Mix Measurement Error

The impact of measurement error in patent case-mix variables (e.g., comorbidities) on estimation of provider effects and SRR and on the ability to correctly identify truly extreme providers was examined in detail by Senturk *et al*. [33] for FE model (2) as well as CMS RE model (1).

As expected, and similar to results in classical measurement error for generalized linear models [68], estimates of provider effects $\left\{\gamma_{i}\right\}$ are biased as a function of increasing levels of measurement error for both FE and RE models. However, for the RE model the bias is compounded due to shrinkage. (See earlier discussion in Section 2). Although measurement error does not affect the estimation of SRR on average, measurement error does contribute to higher variability in SRR. With respect to both FE and RE profiling models, measurement error does negatively impact the ability to flag truly under-performing providers and the reduction in performance depends on the case-mix effect size (β). Finally, the work in [33] shows explicitly that for the hypothesis testing procedure used with FE model (2), the coverage probabilities are off (below the 95% target) with case-mix measurement error. Similarly, for the bootstrap approach to obtain 95% CI for SRR to flag extreme providers used with the CMS RE model (1), the average CI length is increased with measurement error. See [33] for details.

### 6.4. FE Model Estimation and Inference under Highly Sparse Outcome

Recently, Estes *et al*. [45] examined the feasibility of estimation and inference for FE model (2) under extremely sparse outcome, termed “low information” context and proposed a correction method to address the instability in FE model estimation in the low information context. A motivation for this is that cause-specific hospitalization in ESKD patients, such as dialysis access infection-related is low. Even for all infection-related hospitalizations, the prevalence is only about 8% compared to > 30% for call-cause 30-day readmission. Thus, it is of interest to further understand the performance of FE model under highly sparse patient outcomes.

**Figure 8:**
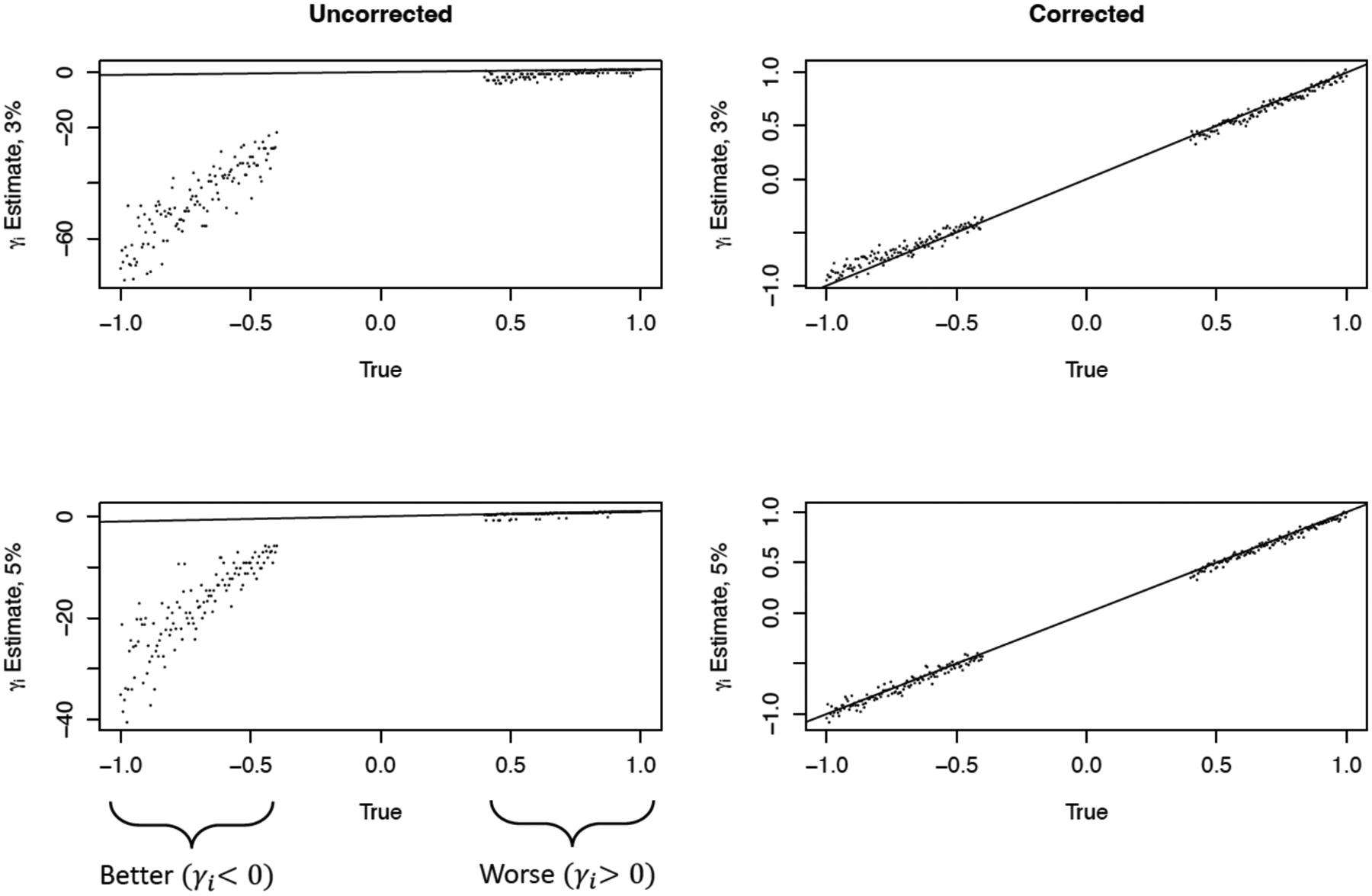
Instability in estimation of provider effects (left) and corrected estimates (right). Adapted from [45].

Figure 8 illustrates the main findings with respect to estimation of facility/provider effects $\gamma_{i}$’s. For highly sparse outcomes (e.g., at 3% and 5%) FE estimates of facility effects for truly over-performing $\left(\gamma_{i}<0\right)$ facilities are particularly unstable. A bias-corrected estimation procedure, similar to Firth’s correction [69, 70], was effective at stabilizing estimation of $\left\{\gamma_{i}\right\}$ (Figure 8 - right side). Note that the unstable estimates are in the same direction as the true effects. Also, even though the provider effects (for $\gamma_{i}<0$) are unstable, corresponding estimates for SRR are reasonably well estimated. See [45] for details.

**Figure 9:**
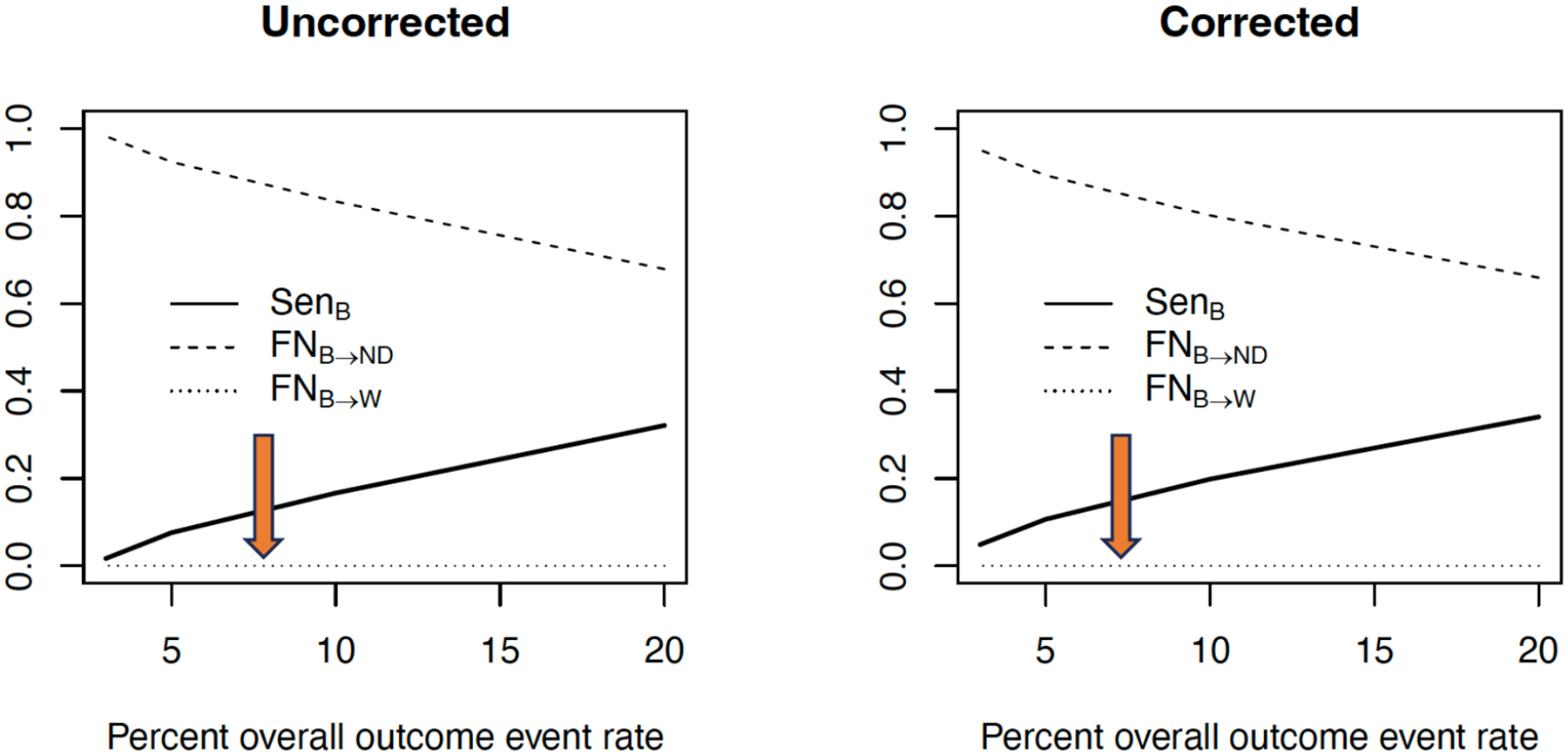
Sensitivity (Sen) of correctly flagging “better” (B) providers and false negative classification of B providers as not different (ND) and as worse (W) providers as a function of outcome sparsity (outcome event rate: 5% to 20%). Adapted from [45].

Finally, we summarize the impact of sparse data on misclassification or misidentification of providers that perform better than expected relative to the reference norm. As discussed earlier, the main objective in profiling with respect to regulatory or payment reimbursement focuses on flagging (classifying) providers that performs worst (W) than expected and providers whose performances are not different (ND) from the reference norm. Thus, providers that perform better (B) than expected are not “relevant” with respect to current payment policy or regulatory objectives because the policy does not incentivize “top” performers. Therefore, it is of interest under a policy regime that does not incentivize (distinguish) B providers, to understand the flagging behavior of FE model under sparse outcome. Figure 9 summarizes this result. The following observations can be made from Figure 9: (a) The false negative rate of misclassifying a B provider as a W provider is zero $\left(FN_{B\to W}=0\right)$; that is, no better provider is misclassified as a worse provider. This is not surprising since W and B providers are on the opposite tails of the distribution of providers. (b) It is not uncommon for false negative classification of a B provider as a ND provider $\left(FN_{B\to ND}\right).\;FN_{B\to ND}$ is common for the extremely low information context (e.g., 3%, 5% outcome event rate) and its decreases as the outcome event is more prevalent. See [45] for an extended discussion.

### 6.5. Discussion, Extensions and Future Research

The selected studies described above in Section 6 examining the operating characteristics of FE profiling model and also comparison with the RE model have shed light on several important limitations and model assumptions as well as providing guidance for practice. The seminal work of Kalbfleisch and Wolfe [30] elucidated the overall performance of RE and FE with respect to overall average error and pointed out that FE have smaller error for extreme providers of interests and also that RE models structure leads to biased estimates in the presence of confounding between patient risk factors and provider effects. In the contexts of outcomes for the ESKD patients, such as hospital readmission, where the rates are high, the need to stabilize estimation due sparse outcomes is not necessary. Chen *et al*. [31] illustrate that with outcome rate between 14%−40%, the CMS RE model does poorly in identifying under-performing providers compared to the FE model. This work also quantified the impact of model choice (FE vs. RE) compared to other important aspects of profiling analysis, specifically adequate case-mix adjustment. With respect to the ability to identify truly under-performing providers, the impact of model choice can be as large as the impact of extremely poor case-mix adjustment.

For extremely sparse outcomes, FE model estimation of provider effects, especially for over-performing providers, are unstable [45], although this has limited effect on provider-specific SRR estimates (the quantity of interest reported in profiling analysis). This study suggests that the FE profiling model, even uncorrected, is still useful in the low information context with respect to the current public policy goal of identifying W and ND providers. However, if the public policy goal evolves to also incentivize for better performance, then novel methods able to correctly identify B providers with high sensitivity are needed.

Finally, we also note that it would be more instructive in future comparative studies to include as close as possible to currently adopted models used in practice (e.g., CMS models) so that conclusions have more direct bearing on current practice.

## 7. CONCLUSION AND DISCUSSION

Although the idea of evaluating or monitoring health care providers dates back more than a century [12], various profiling approaches were increasingly applied in different settings in the mid 1980s to 2000s, including schools [71, 72], individual surgeons [73], and health care providers more generally [19, 74]. Particularly in the US, there is a renewed research interest on profiling methodologies and their applications in monitoring health care providers after the launch out of Hospital Compare [29] and the evaluation of the RE methodology adopted by CMS [24]. These endeavors have broad societal impacts, ensuring patient health care quality and safety, improving accountability, and eliminating disparity in health outcomes across populations.

In this work, we have focused on several selective FE profiling approaches used in the ESKD population, discussed their rationale, and situated these works within the larger profiling literature (see Figure 1), including RE profiling approaches. In doing so, we have focused on several limitations, assumptions and advantages of FE profiling model and the situations where they are preferred to the commonly applied RE models. We have also highlighted the need for critical evaluation of the contexts and rationale appropriate for the choice of models to use. Some aspects are statistical considerations (outcome sparsity, adequate case-mix adjustment etc.) while other aspects focus on the broader objectives of profiling. This includes the rationale for exclusion of specific risk adjustment factors, such as race and socioeconomic status, to support a policy objective to reduce health outcome disparity; uses of time-dynamic profiling for longer-term longitudinal outcomes in the ESKD population in order to focus on the continuity of care and longer-term patient outcomes; and profiling model attributing 30-day hospital readmission to dialysis facilities to drive policy objectives to increase coordination between discharging hospitals and dialysis facilities.

Also, we have highlighted several open areas for future research and hope that this review contributes to new researchers interested in applying profiling methods or developing new methodologies.

## Figures and Tables

**Table 1:** Past-Year Comorbidities Significantly Associated with Odds of 30-Day Readmission from Time-Dynamic Profiling (TDP) Model

Past-year comorbidities	OR	95%CI	
High-risk index hospitalization	1.52	1.39	1.66
Drug and alcohol disorders	1.37	1.22	1.55
End-stage liver disease	1.32	1.16	1.49
Transplants	1.10	1.01	1.19
Metastatic cancer/acute leukemia	1.27	1.18	1.37
Severe hematological disorders	1.18	1.03	1.34
Pancreatic disease	1.27	1.10	1.48
Seizure disorders and convulsions	1.21	1.02	1.44
Severe Cancer	1.16	1.02	1.33

OR = odds ratio; CI = confidence interval; other patient risk adjustment factors included in the TDP model: age; sex; BMI; cause of ESKD: diabetes; amputation status; coronary obstructive pulmonary disease; cardiorespiratory failure/shock; coagulation defects and other specified hematological disorders; fibrosis of lung or other chronic lung disorders; hemiplegia/paraplegia/paralysis; hip fracture/dislocation; other infectious disease and pneumonias; other cancers; psychiatric comorbidity; respirator dependence/tracheostomy status; rheumatoid arthritis and inflammatory connective tissue disease; septicemia/shock; severe infection; ulcers.

## APPENDIX

Inference: Flagging Extreme Providers under RE Model (1) - Bootstrap CI for SRR

Under RE model (1), the procedure to flag extreme providers is based on the following bootstrap CI approach from [16, 24].

Step 0: Fit the generalized linear mixed effects RE model (1) to obtain provider-specific REs ${\overset{ˆ}{\gamma }}_{i},i=1,\ldots ,I$, overall mean ${\overset{ˆ}{\gamma }}_{0}$, variance ${\overset{ˆ}{\sigma }}^{2}$, and patient case-mix coefficients $\overset{ˆ}{\beta }$. Substitute these estimates into (3) to calculate $S{\overset{⏜}{RR}}_{i}$. SAS PROC GLIMMIX [75] or R library Ime4 [76] function glmer, can be used to fit the model. The CMS implementation uses SAS PROC GLIMMIX [16, 18, 24].

Step 1: Generate a bootstrap dataset by sampling I providers with replacement from the original dataset. Denote the unique set of providers sampled by ${\tilde{I}}^{\left(b\right)}$, where b indexes a bootstrap dataset.

Step 2: For bootstrap dataset b, fit mode1 (1) as in step 0, treating each resampled provider as distinct. Compute ${\overset{ˆ}{\beta }}^{\left(b\right)},{\overset{ˆ}{\gamma }}_{0}^{\left(b\right)},{\overset{ˆ}{\sigma }}^{2(b)}$, and provider-specific effects and variances $\left\{{\hat{\gamma }}_{i}^{\left(b\right)},{\hat{\text{Var}}}^{\left(b\right)}\left(\gamma_{i}\right),i=1,\ldots ,I\right\}$.

Step 3: Generate a provider RE from the provider-specific distribution from step 2 for each unique provider sampled in step 1. The posterior distribution of each RE is approximated by ${\hat{\gamma }}_{i}^{(b)*}\sim N\left({\hat{\gamma }}_{i}^{\left(b\right)},{\hat{\text{Var}}}^{\left(b\right)}\left(\gamma_{i}\right)\right)$.

Step 4: Calculate estimates of $SRR_{i}$ for each unique provider i sampled in step $1,i\in {\tilde{I}}^{\left(b\right)}$:

$${\hat{SRR}}_{i}^{\left(b\right)}=\sum_{j=1}^{N_{i}}{\hat{p}}_{ij}^{\left(b\right)}/\sum_{j=1}^{N_{i}}{\hat{p}}_{ij,M}^{\left(b\right)}=\sum_{j=1}^{N_{i}}g^{-1}\left({\hat{\gamma }}_{i}^{\left(b\right)*}+Z_{ij}^{T}{\hat{\beta }}^{\left(b\right)}\right)/\sum_{j=1}^{N_{i}}g^{-1}\left({\hat{\gamma }}_{0}^{\left(b\right)\text{*}}+Z_{ij}^{T}{\hat{\beta }}^{\left(b\right)}\right).$$

Step 5: Repeat bootstrap sampling and estimation, step 1–4, 500 times (b=1,…,500). Form 95% CI for $SRR_{i}$ for each provider i=1,…,I.

### Inference: Flagging Extreme Providers under FE Model (2) – Hypothesis Testing

For the FE profiling model (2), flagging extreme providers is based on testing the null hypothesis $H_{0}:\gamma_{i}=\gamma_{M}$ or equivalently $SRR_{i}=1$ for provider i as proposed in [38]. FE model (1) can be fitted using the iterative one-step Newton-Raphson algorithm outline above; for details see [38].

Step 1: Fit FE model (1) and fix the parameters β and $\gamma_{M}$ at their estimated values $\overset{ˆ}{\beta }$ and ${\overset{ˆ}{\gamma }}_{M}$.

Step 2: For provider i, draw B=500 samples under the null hypothesis, ${\left\{Y_{ij}^{\left\{b\right\}}:j=1,\ldots ,N_{i}\right\}}_{b=1}^{B}$.

Each sample is drawn from the Bernoulli distribution with trial probability $\text{exp}\left({\overset{ˆ}{\gamma }}_{M}+Z_{ij}^{T}\beta \right)/\left(1+\text{exp}\left({\overset{ˆ}{\gamma }}_{M}+\right.\right.\left.Z_{ij}^{T}\beta \right)$).

Step 3: Calculate the number of outcome events (e.g., 30-day readmissions) in the resampled data as $Y_{i.}^{\left(b\right)}=\sum_{j=1}^{N_{i}}\;Y_{ij}^{\left(b\right)}$.

Step 4: Calculate the p-value for testing $H_{0}:\gamma_{i}=\gamma_{M}$ for provider i follows. Compute ${SL}_{i}^{+}=\sum_{b=1}^{B}\;\left[0.5I\left(Y_{i}^{\left(b\right)}=\right.\right.\left.\left.O_{i}\right)+I\left(Y_{i}^{\left(b\right)}>O_{i}\right)\right]$, where $O_{i}$ denotes the number of outcome events for provider i in *original* dataset and I(A) denotes the indicator function for event A. Similarly, compute ${SL}_{i}^{-}=\sum_{b=1}^{B}\;\left[0.5I\left(Y_{i}^{\left(b\right)}=O_{i}\right)+\right.\left.I\left(Y_{i}^{\left(b\right)}<O_{i}\right)\right]$. The p-value for testing $H_{0}$ is $p=2\times \text{min}\left\{SL_{i}^{+},SL_{i}^{-}\right\}$.

Step 5: Steps 2–4 are repeated for each provider i=1,…,I.

Note that for the log-linear profiling model (6) for count/rate outcomes [34], the test statistics is $T_{i}=\sum_{j=1}^{N_{i}}\;{\overset{ˆ}{\mu }}_{ij}$ and the above procedure can be used where under the null $Y_{ij}^{\left(b\right)}\sim \text{Pois}\left(\text{exp}\left\{\text{log}\left(t_{ij}\right)+{\overset{ˆ}{\gamma }}_{M}+\right.\right.\left.Z_{ij}^{T}\overset{ˆ}{\beta }\right\}$ and $O_{i}$ replaced by $T_{i}$, and $Y_{i}^{\left(b\right)}$. replaced by $T_{i}^{\left(b\right)}=\sum_{j=1}^{N_{i}}\;\text{exp}\left\{\text{log}\left(t_{ij}\right)+{\overset{ˆ}{\gamma }}_{i}^{\left(b\right)}+Z_{ij}^{T}\overset{ˆ}{\beta }\right\}$.

## LIST OF ABBREVIATIONS

BMI: body mass index
CI: confidence interval
CMS: Centers for Medicare and Medicaid Services
COPD: chronic obstructive pulmonary disease
COPSS: Committee of Presidents of Statistical
CV: cardiovascular
eGFR: estimated glomerular filtration rate
ESRD: end-stage renal disease
ESKD: end-stage kidney disease
LME: linear mixed effects
MI: myocardial infarction
MVCM: multilevel varying coefficient model
NIDDK: National Institute of Diabetes and Digestive and Kidney Diseases
NS: not significant
NHS: National Health Service (United Kingdom)
OR: odds ratio
RAE: recurrent adverse event
SDRR: standardized dynamic readmission ratio
SER: standardized event ratio
SMR: standardized mortality ratio
SRR: standardized readmission ratio
SW: significantly worse
UM-KECC: University of Michigan Kidney Epidemiology and Cost Center
USRDS: United States Renal Data Systems
